# Centrifugal Microfluidic Cell Culture Platform for Physiologically Relevant Virus Infection Studies: A Case Study with HSV-1 Infection of Periodontal Cells

**DOI:** 10.3390/bios14080401

**Published:** 2024-08-20

**Authors:** Juliane Fjelrad Christfort, Morgane Ortis, Hau Van Nguyen, Robert Marsault, Alain Doglio

**Affiliations:** 1 MICORALIS (E.A. 7354), Faculty of Dental Surgery and Odontology, University Côte d’Azur, 06300 Nice, France; morgane.ortis@univ-cotedazur.fr (M.O.); robert.marsault@univ-cotedazur.fr (R.M.); 2 IDUN Centre of Excellence, Department of Health Technology, Technical University of Denmark, 2800 Kgs. Lyngby, Denmark; havng@dtu.dk

**Keywords:** organ-on-a-chip, centrifugal microfluidics, dynamic viral infection, infection gradient, periodontal tissues, human herpes viruses, acyclovir

## Abstract

Static well plates remain the gold standard to study viral infections in vitro, but they cannot accurately mimic dynamic viral infections as they occur in the human body. Therefore, we established a dynamic cell culture platform, based on centrifugal microfluidics, to study viral infections in perfusion. To do so, we used human primary periodontal dental ligament (PDL) cells and herpes simplex virus-1 (HSV-1) as a case study. By microscopy, we confirmed that the PDL cells efficiently attached and grew in the chip. Successful dynamic viral infection of perfused PDL cells was monitored using fluorescent imaging and RT-qPCR-based experiments. Remarkably, viral infection in flow resulted in a gradient of HSV-1-infected cells gradually decreasing from the cell culture chamber entrance towards its end. The perfusion of acyclovir in the chip prevented HSV-1 spreading, demonstrating the usefulness of such a platform for monitoring the effects of antiviral drugs. In addition, the innate antiviral response of PDL cells, measured by interferon gene expression, increased significantly over time in conventional static conditions compared to the perfusion model. These results provide evidence suggesting that dynamic viral infections differ from conventional static infections, which highlights the need for more physiologically relevant in vitro models to study viral infections.

## 1. Introduction

During the past decades, microfluidics has gradually been more applied to create dynamic and physiologically relevant in vitro cell models [1,2]. The term microfluidics refers to the technology of manipulating small fluid volumes through micro-sized channels, chambers, or wells, enclosed or inserted into a microdevice, referred to as a chip [2]. This principle has been employed as a novel method to culture 2D and 3D cell models in perfusion, resulting in more in vivo-like cell cultures compared to the traditional static methods, such as well plates. The microfluidic cell culture technology has been used to study and mimic a variety of different cell types, tissues, and organs, such as the brain, intestine, liver, and kidney, including for organ-on-a-chip purposes [1,3,4,5]. However, most microfluidic systems require external pumps and tubing to generate and maintain perfusion, which makes them complex and labor-intensive to implement.

More recently, centrifugal microfluidics has been applied as an alternative technology to cultivate cells [6,7]. Here, centrifugal forces are used to manipulate fluids through channels and chambers in a disc-shaped device when it is rotated. Compared to conventional laminar microfluidic chips, the risk of cell damage due to different forces and an unprecise flow (for example, centrifugal, Coriolis and Euler forces, and Dean flow as a result of the spiral shape [8]) could present a challenge when applying centrifugal microfluidics for cell culturing. However, advantages of centrifugal microfluidic technology include its simplicity and ease of use, since microchannels, chambers, and other microfluidic components are integrated into a compact disc and there is no need for external pumps, valves, or tubing [9]. While this can limit the volumes for fresh culture medium and waste, it is highly advantageous in cell culture, where all components need to be thoroughly sterilized before use.

To study viral infections in vitro, cells cultured in well plates remain the gold standard model. However, static monolayer cell models do not adequately mimic the complex environment of the human body [10,11]. Specifically in virology, more dynamic in vitro platforms are crucial for improved disease modeling. So far, the understanding of infection kinetics, host-virus interactions, and drug resistance has been limited by the inability to reproduce the dynamic complexity of viral infections in vitro [12]. To meet this need, microfluidic platforms have been investigated to recapitulate most life cycle steps of different pathogenic viruses, including a 3D liver culture for human hepatitis B virus [13], a gut-chip prototype for human enterovirus infection [14], and a nervous system-on-a-chip enabling pseudorabies virus infection [15]. Recently, microfluidic chips have been proposed to investigate different strains of influenza or coronaviruses responsible for pandemic outbreaks [16] or epidemic viruses such as Ebola [17].

Despite oral health being a major global health issue [18] and accumulating evidence associating the oral microbiota with several systemic diseases [19,20,21,22,23,24], only a few oral microfluidic cell culture platforms have been reported [25]. A recent review (2024) includes six original research articles describing microfluidic chips mimicking soft tissues in the oral cavity, including tissue models with one or more cell types [26]. For example, Rahimi et al. [27] developed a 3D tissue model with oral keratinocytes and fibroblasts cultured in collagen in polydimethylsiloxane microchannels. The model was applied to investigate cellular responses to dental materials or oral bacteria and found more sensitive when evaluating cell viability than traditional well-plate cultures [27]. Apart from soft tissues, microfluidic models of the oral cavity also include models mimicking teeth, bones, cancer, and salivary glands [26].

**Figure 1:**
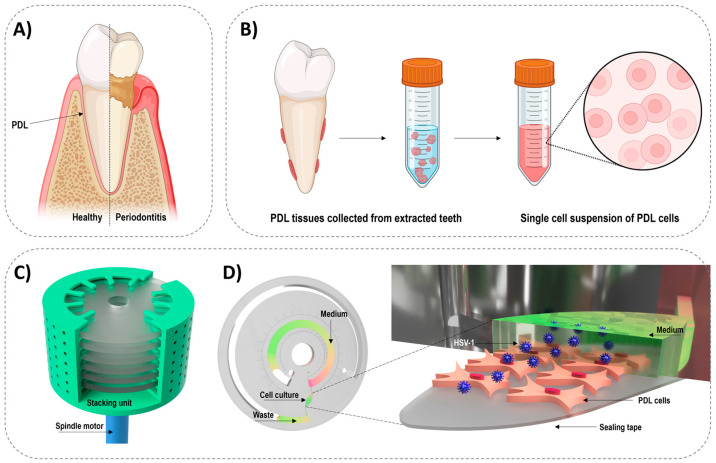
Overview of the study background and setup. (**A**) Illustration of the periodontal environment in health and diseases, showing the location of the PDL between the tooth root and the alveolar bone. (**B**) A simplified schematic overview of the isolation of single PDL cell suspensions from the PDL tissues surrounding extracted teeth. (**C**) Illustration of the stacking unit with multiple chips inside installed on a spindle motor (see Figure S1 in Supplementary for additional illustrations and pictures of the stacking unit). (**D**) Illustration of the microfluidic chip design (**left**), where a zoom shows the location of the cells in the cell culture chamber and the concept of introducing viruses with flow (**right**).

Among oral diseases, periodontitis is a widespread chronic gum disease, advancing in relapses and successive stages over years. It leads to progressive destruction of the tooth supporting tissues [28,29], including the gingiva, the alveolar bone, and the periodontal dental ligament (PDL) (Figure 1A). Traditionally, the pathogenesis of periodontitis has been explained as an infection caused by pathogenic oral bacteria colonizing the tooth surface and the gingival sulcus [30]. However, bacterial-host interaction alone does not adequately explain the clinical characteristics of the disease (e.g., its progressive nature and localized occurrence) [31]. Recently, human herpes viruses have been suggested to play a major role in the progression of periodontitis [20,32,33], and numerous studies have shed light on the potential involvement of human herpes simplex type 1 (HSV-1) in the pathogenesis of periodontitis [34,35]. Recently, insights from our lab provided the first evidence supporting efficient HSV-1 infection of PDL cells [36]. The PDL is a thin connective ligament tissue connecting the tooth root to the dental alveolar bone (Figure 1A), and PDL cells have been shown to synthesize immunomodulatory cytokines influencing the local response to infections [37,38,39,40]. Thus, the PDL tissue plays a crucial role in periodontal integrity and homeostasis.

In the present work, we establish a microfluidic cell culture model of primary human PDL cells isolated from tissue surrounding freshly extracted teeth (Figure 1B) to study interactions between human cells and periodontal pathogens, such as HSV-1, involved in periodontal diseases. As a major novelty, the model is based on centrifugal microfluidics, which has only recently been applied for long-term (6 days) mammalian cell culture [41], but never with primary cells or for virus infection studies. Additionally, to increase the throughput of the system, we propose a 3D-printed stacking unit to run multiple replicates on the same motor (Figure 1C and Figure S1 in Supplementary). Thereby, we aim to accommodate the need for a dynamic model to study viral infections in a more physiologically relevant—and yet simple—manner, allowing for real-time analysis of the inflammatory response of cells upon viral challenge.

## 2. Materials and Methods

### 2.1. Design and Fabrication of the Centrifugal Microfluidic Cell Culture Platform

The platform includes a brushless spindle motor (Maxon, Switzerland) (Figure 1C) and a microfluidic disc-shaped chip for cell culture (Figure 1D) [41]. When spinning the disc, a flow of growth medium is created in the microfluidic channels. The chip comprises a cell chamber (23 µL, 0.30 cm^2^) and a medium (3 mL) and waste chamber (5 mL), which are all connected via microchannels (0.15 mm) (Figure 1C). The microchannels between the cell chamber and the medium and waste chambers are placed in the top of the cell chamber to ensure minimal disturbance of the cells during perfusion. Additionally, these microchannels act as passive valves, preventing overflow of cells into the spiral-shaped media chamber when cells are introduced into the chip. The openings (venting holes) of the medium and waste chamber were closed with sterile adhesive filters (HJ-bioanalytik, Erkelenz, Germany).

The microfluidic chip was designed and fabricated as previously described [41]. Briefly, the chip was designed in the computer-aided software SolidWorks 2018 (Dassault Systèmes, Vélizy-Villacoublay, France) and assembled from four layers of poly(methyl methacrylate) (PMMA) (0.5 and 5 mm) (PSC A/S, Brønderslev, Denmark; Nordisk plast, Randers, Denmark) bonded together by three layers of double-sided pressure-sensitive adhesive (PSA) (ARcare^®^ 90106, Adhesive Research, Limerick, Ireland). The PMMA layers were fabricated using a laser ablation technique (Epilog Mini 18 30 W system, Epilog, Golden, CO, USA), and the PSA was cut with a Graphtec cutter (CE-40, Graphtec, Irvine, CA, USA) with a bonding press (PW 10 H, P/O/Weber, Remshalden, Germany) with a force of 1 kN for 1 min. The stacking unit was fabricated using a 3D printer (Formlabs, Somerville, MA, USA).

### 2.2. Preparation of PDL Single Cell Suspensions and Cell Culture

Human primary PDL cells were isolated from PDL tissue surrounding extracted teeth (Figure 1B) and immortalized as previously described [36]. All patients were informed of their right to oppose the use of their specimens and data for research purposes (biomedical collection N° DC-2022-5040, French Ministry of Higher Education, Research and Innovation, Paris, France). Briefly, extracted teeth were immersed in phosphate-buffered saline (PBS) containing antibiotics and antifungals and kept at 4 °C for less than 24 h. After 2 washings in PBS, PDL tissues were collected through scalpel-scraping of the mid-third of the root surface. A single cell suspension of PDL cells was obtained after digestion of PDL tissues with type I collagenase (3 mg/mL) and dispase II (4 mg/mL) for 30–40 min at 37 °C and passed through a cell strainer (70 µm). After centrifugation, the cells were resuspended in alpha minimum essential medium (αMEM) supplemented with 0.292 µg/mL L-Glutamine and 10% fecal calf serum (FCS) [36].

For regular cell culture, the PDL cells were cultured in αMEM containing 10% (*v*/*v*) FCS, 100 units/mL penicillin, and 100 µg/mL streptomycin and maintained in a humidifying incubator (37 °C, 5% CO_2_). Between passages, the cells were dissociated with a trypsin/EDTA solution (0.25%/0.02%) and subcultured in complete medium. The cells were evaluated with a bright-field microscope (Nikon TMS, Nikon, Tokyo, Japan) and counted using a Malassez counting chamber (Marienfeld Superior, Lauda-Königshofen, Germany).

### 2.3. PDL Cell Seeding and Growth in the Microfluidic Chip

Prior to cell seeding, the fully assembled chip was cleaned and sterilized for 1 h with 0.5 M sodium hydroxide and carefully rinsed with sterile water. The cell culture chamber was coated with 23 µL of Matrigel^®^ Matrix (Corning Inc.^®^, Corning, NY, USA) at 37 °C for 1 h, after which the remaining Matrigel was removed. The PDL cells were seeded by adding a suspension of cells in αMEM directly to the cell chamber using a syringe needle (7.7 × 10^4^ cells/cm^2^). The cell suspension volume was limited to 23 µL, so it did not overflow into the spiral channel. The microfluidic chips were placed in the stacking unit on the motor, which was placed inside an incubator traditionally applied for cell culture (37 °C with 5% CO_2_ in a humidified atmosphere). The cells were allowed to adhere in static conditions overnight before the medium chamber was filled with cell culture medium and rotation was initiated (0.7 Hz). The resulting flow rate was determined by measuring the movement of the cell culture medium over a defined period of time. By using volume markers for every 20 and 100 µL along the medium chamber (Figure S2 in Supplementary), the difference in medium level could be determined with sufficient accuracy. After 24 and 48 h, the motor was briefly stopped and the cells were imaged (ZEISS Axio Vert.A1, ZEISS, Oberkochen, Germany).

### 2.4. HSV-1 Infection of PDL Cells in the Microfluidic Chip

The HSV-1 isolate used in this study was a mCherry red fluorescent protein-tagged HSV-1 strain (HSV-1 mCherry) [42]. HSV-1 viral stock was produced in αMEM medium using PDL cells as amplifying cells. The viral titer of the stock was determined by testing serial viral dilutions to define the 50% tissue culture infectious dose that promoted a cytopathic effect on PDL cells.

When the cells had grown to a confluent monolayer in the disc (3 days after seeding), the motor was briefly stopped, a suspension of HSV-1 mCherry in αMEM was filtered (0.4 µm) and added to the medium chamber (multiplicity of infection (MOI) of 6), and the rotation was reinitiated to allow the virus to reach the PDL cells via flow. After 1.5 h, the cells were carefully washed with PBS, and fresh αMEM was added. For studies in the presence of the antiviral drug acyclovir, it was added to the medium (40 µg/mL) when introducing HSV-1. At 2, 24, and 48 h post infection, the motor was briefly stopped, and the chip was taken to the microscope (ZEISS Axio Vert.A1, ZEISS, Oberkochen, Germany) for brightfield and fluorescent imaging. Immediately after imaging, the chip was placed back on the motor for continuous spinning.

For comparison to static conditions, the same study was carried out with PDL cells cultured in a 96-well plate. To mimic the conditions in the chip as closely as possible (except for the flow), all protocol steps, procedures, cell seeding, and chemicals were kept constant. 96-well plates were applied as static references since each well has approximately the same surface area as the cell culture chamber in the chip (0.32 cm^2^ in the 96-well compared to 0.30 cm^2^ in the chip).

### 2.5. RNA Extraction and RT-qPCR

At 2 h and 48 h post infection, the PDL cells were detached directly in the chip using a lysis buffer (Buffer RLT, Qiagen, Hilden, Germany) and collected with a syringe needle for quantitative reverse transcription polymerase chain reaction (RT-qPCR) analysis. RNA extraction was performed using a Qiagen RNeasy Mini Kit (Qiagen, Hilden, Germany) and quantified using a microvolume spectrophotometer (SimpliNano™ Biochrom, Cambridge, UK). All samples were normalized to 1.0 ng/µL in the retro-transcriptase step, which was completed independently of the PCR. RT-qPCR experiments were performed using QuantStudio™ 5 (Applied Biosystems™, Waltham, MA, USA) with 2 ng of cDNA (equivalent RNA), using Power SYBR^®^ Green PCR Master Mix (Qiagen, Hilden, Germany), in a final volume of 20 µL. The following amplification conditions were applied: 95 °C, 10 min; (95 °C, 15 s; 60 °C, 1 min) cycled 40 times. The following six target genes were quantified: infected cell protein (ICP) 0, 4, and 8, and the antiviral proteins interferon (IFN) α, β, and λ. Each sample was run in technical triplicates using specific primer sets for each gene (Table 1). Relative gene expression levels were calculated using the 2^−ΔΔCT^ method, with the glyceraldehyde 3-phosphate dehydrogenase (GAPDH) gene as the reference gene and corresponding controls (PDL cells without HSV-1 infection (with/without acyclovir)) as reference samples.

### 2.6. Data Analysis and Visualization

Visualization and analysis of the data were carried out in GraphPad Prism version 10.0 (GraphPad Software Inc., La Jolla, CA, USA). Other graphical illustrations were created using Biorender.com. For averages, the results are shown as mean ± standard deviation.

## 3. Results and Discussion

### 3.1. Centrifugal Microfluidic Chip Design

The applied microfluidic chip is based on centrifugal microfluidics, which allows for cell culturing in perfusion without the need for external pumps or tubing. The culture chamber is perfused with a liquid flow going from the medium chamber to the waste chamber. In general, the microfluidic chambers and channels in the chip were designed and architected to minimize Dean flow in the cell growth chamber, preventing the occurrence of secondary flow and maintaining laminar flow where the cells are grown. The medium chamber, with its spiral shape, risks being exposed to Dean flow. However, in the cell growth chamber, Dean flow is minimized due to the oval shape and orientation following the path of the centrifugal force generated by the spinning motor. This setup allows the solution to be pumped through the cell growth chamber by centrifugal force, resulting mainly in laminar flow.

When characterizing the flow of cell culture medium in the chip, a flow rate of 0.23 ± 0.14 µL/min (n = 14) was achieved when applying a rotational frequency of 0.7 Hz. At this flow rate, no damage to the cells was observed by microscopy. Additionally, comparing static and perfusion cell culture conditions did not reveal any differences in cell morphology and gene expression. For example, the housekeeping gene GAPDH was detected at very comparable levels by RT-qPCR in both conditions. This rotational frequency was applied throughout the present work.

The observed flow rate of 0.23 ± 0.14 µL/min is similar to previously reported values, where a rotational frequency of 0.7 Hz corresponded to a flow rate of 0.25 µL/min [41]. In the same study, a good linear dependency (r^2^ = 0.97) was observed from 0.7–1.0 Hz. Additionally, the maximum shear stress was simulated to be 0.08 mPa (at 0.6 µL/min) [41]. A shear stress in this range should not result in any adverse effects on the cells during the experiment [43]. In any case, the expected shear stress from a flow rate around 0.25 µL/min, as used in the present work, is estimated to be well below any value stressing or damaging the cells.

### 3.2. Culturing of Periodontal Ligament Cells in the Microfluidic Chip

**Figure 2:**
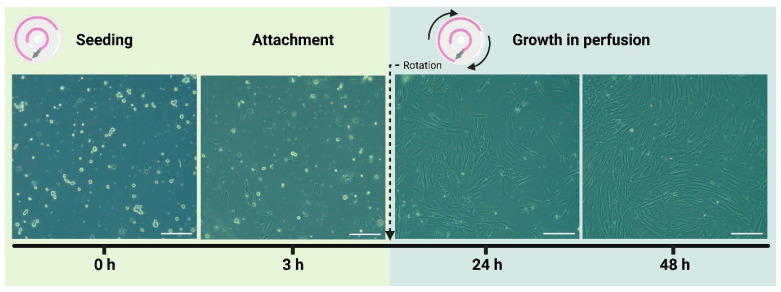
Seeding and growth of PDL cells in the microfluidic chip device. Representative brightfield images (10×) at 0 h (immediately after seeding), at 3 h (after attachment in static conditions), and at 24 h and 48 h (after growth in perfusion). A total of 7.7 × 10^4^ cells/cm^2^ were seeded at 0 h. All scale bars represent 200 µm.

Based on the PDL cell model presented by Chevalier and Ortis et al. [36], single-cell suspensions of primary PDL cells were obtained from PDL tissues collected from extracted teeth. Following isolation, PDL cells were seeded in the cell culture chamber of the chip and allowed to attach in static conditions overnight. After attachment, the cells grew and formed an 80–100% confluent monolayer in the cell culture chamber after 48 h perfusion (Figure 2). In total, the PDL cells were successfully cultured in the chip for 5 consecutive days (3 days + 2 days infection study) in perfusion.

The morphology of the growing and confluent primary PDL cell culture was similar to that of fibroblast-like cells, which has also been observed for primary PDL cells cultured in static conditions (Figure S3 in Supplementary and [36]).

Previously, the present chip has been used to successfully culture commercially available adherent cell lines within a similar time frame to that of the present study (Caco-2 and HeLa cells during 24 h and 6 days, respectively) [41], where we, in the present work, further adapt the method to allow culturing of primary cells directly isolated from fresh human tissue.

### 3.3. HSV-1 Infection of PDL Cells and Treatment with Acyclovir

**Figure 3:**
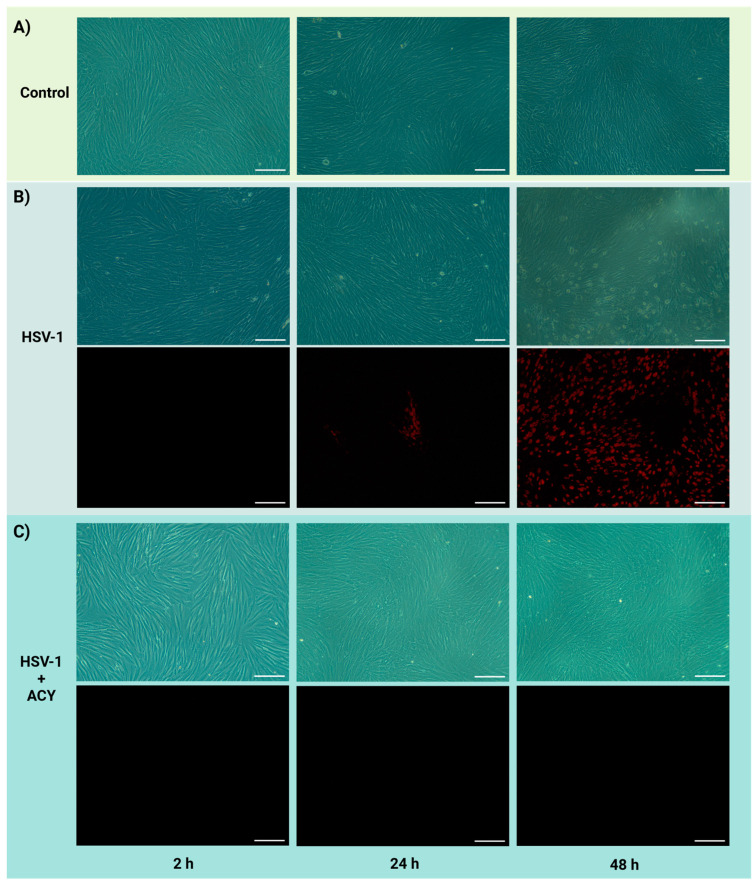
HSV-1 infection of PDL cells in perfusion. Representative corresponding brightfield and fluorescent images (10×) showing (**A**) the growth of PDL cells without HSV-1 infection (control), (**B**) HSV-1 infection of PDL cells (MOI 6), and (**C**) HSV-1 infection of PDL cells (MOI 6) in the presence of acyclovir (40 µg/mL). The cells were imaged at 2, 24, and 48 h after adding HSV-1 to the cell culture medium. All scale bars represent 200 µm.

To investigate how flow affects the establishment and treatment of HSV-1 infections in PDL cells, PDL cells were infected with HSV-1 mCherry (Figure 3). In contrast to traditional virus infection protocols, HSV-1 was added to the medium in the medium chamber and, hereafter, allowed to reach the cells with the perfusing medium.

No visual effect could be seen on the cells 2 h post infection when compared to cells without infection, and no fluorescent signal was observed from HSV-1 mCherry (Figure 3A,B). After 24 h, the first signs of viral infection were visible by fluorescent imaging, but no remarkable morphological changes, revealing HSV-1 cytopathic effect, could be observed. After 48 h, the infection increased in intensity, and most cells were infected. Profound morphological changes, such as ballooning and detachment of dead cells (Figure 3B), highlighted that the cytolytic activity of HSV-1 in PDL cells was maintained in perfusion (see Figure S4 in Supplementary for enlarged images with highlighted characteristics).

Previously, onset of cytopathic effects in primary PDL cells had been observed already after 24 h in static conditions [36]. This may reflect the possibility that, in static conditions, the accumulation of new viruses in culture supernatant could favor abundant reinfection events and more rapid cytopathic effects when compared to a microfluidic system preventing virus accumulation in the supernatant. Additional studies are needed to further investigate this aspect, but it suggests that the use of a microfluidic chip with perfusion can be more relevant to mimic viral infections in the oral cavity, where viruses are infecting cells via the salivary and sulcular flows or the blood stream [44].

To test the efficacy of an antiviral drug as an inhibitor of viral spreading in perfusion, PDL cells were infected with HSV-1 in the presence of acyclovir (Figure 3C), a well-known antiviral drug used to treat HSV-1 infections. The presence of acyclovir, added along with HSV-1 in the medium chamber, fully inhibited HSV-1 infection of PDL-cells adherent on the microchip throughout the duration of the infection study (48 h) (Figure 3C).

### 3.4. RT-qPCR Analysis of HSV-1 Gene Expression and Interferon Response in the Microfluidic Chip and Static Conditions

**Figure 4:**
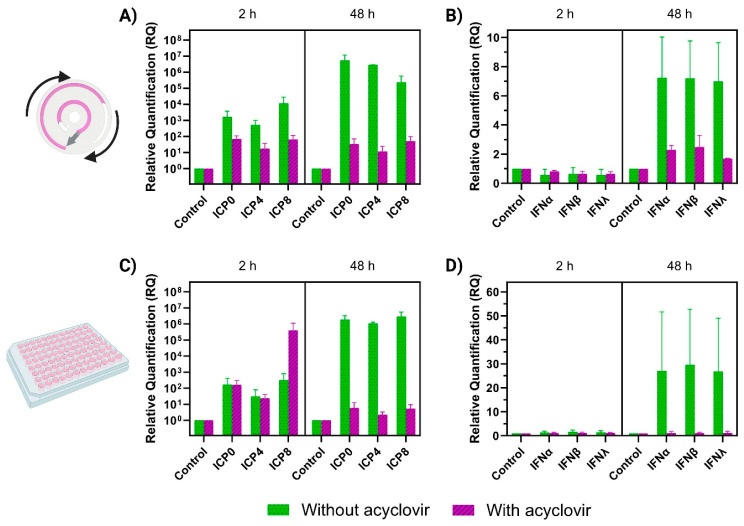
RT-qPCR quantification at 2 and 48 h after HSV-1 infection of PDL cells. Relative quantification of ICP0, ICP4, and ICP8 (**left**) and IFNα, IFNβ, and IFNλ (**right**) after culture in (**A**,**B**) perfusion conditions the chip and (**C**,**D**) static conditions in a 96 well plate. The genes were quantified after HSV-1 infection without acyclovir (green, dotted bars) and with acyclovir (pink, striped bars) (mean ± SD, n = 2–3). All samples were run in technical triplicates, and relative gene expression levels were calculated using the 2−ΔΔCT method and normalized according to the GAPDH as a reference gene and corresponding controls (PDL cells without HSV-1 infection (with/without acyclovir) as a reference sample).

RT-qPCR studies were carried out to provide quantitative information about the viral gene expression and the cellular response to the viral infection (Figure 4). After setting up appropriate methods for RNA collection directly from the cell culture chamber in the chip, we obtained between 10–20 ng/µL RNA from cells in the chip.

HSV-1 infection of PDL-cells in the chip was monitored by analyzing gene expression of the immediate-early (ICP0 and ICP4) and early (ICP8) HSV-1 viral transcripts in the presence and absence of acyclovir. Additionally, expression of the viral transcripts was quantified after a similar experiment carried out in static conditions (96-well plate) to compare dynamic microfluidic-based infection (Figure 4A,B) with infection in static conditions (Figure 4C,D).

In the chip, an increasing level of ICP0, ICP4, and ICP8 was observed during the experiment compared to the control without infection (Figure 4A). Already 2 h post infection, a relative quantification of 1.6 × 10^3^, 5.2 × 10^2^, and 1.2 × 10^4^ was observed for ICP0, ICP4, and ICP8, respectively, and after 48 h, all ICP genes reached higher values (5.3 × 10^6^, 2.8 × 10^6^, and 2.3 × 10^5^, respectively). When comparing the infection to PDL cells cultured in static conditions (Figure 4C), no significant differences were observed, indicating very similar viral gene profiling for the immediate-early and the early HSV-1 viral transcripts in both conditions.

In the presence of acyclovir (Figure 4A), a relative quantification of 7.1 × 10^1^, 1.8 × 10^1^, and 6.6 × 10^1^ was observed for the three ICP genes after 2 h in the chip, which were all lower than the corresponding quantifications without acyclovir. Although not significant, acyclovir appears to have a larger antiviral effect on HSV-1 in the chip (Figure 4A) than in static conditions (Figure 4B) after 2 h, compared to the infection without acyclovir. After 48 h, the presence of acyclovir efficiently inhibited the viral gene expression, which did not increase between 2 h and 48 h and remained much lower compared to infection experiments without acyclovir. Both in static and flow conditions, the presence of acyclovir resulted in remarkedly lower levels of both ICPs after 48 h and was, thus, able to control the HSV-1 infection within the time of the experiment. Acyclovir is known to act by blocking the HSV-1 infection at a late stage of the viral cycle by inhibiting the viral DNA polymerase activity and, thus, only affects viral replication without any effect on viral entry and early viral gene expression [45]. Thereby, it was expected that acyclovir would not have any inhibitory effect on ICP0, ICP4, and ICP8 2 h post-infection.

Concurrently, we investigated the innate cellular response by monitoring expression of interferon genes, namely the type I IFNs (IFNα and IFNβ) and type III IFN (IFNλ), which are antiviral proteins produced by cells as the body’s first line of antiviral defense in response to a viral infection [46]. IFNα, IFNβ, and IFNλ (Figure 4B) were initially (2 h) all present in levels similar to the control group, both with and without acyclovir present. However, at 48 h post infection onset, a relative quantification of 7.2, 7.2, and 7.0 was observed for IFNα, IFNβ, and IFNλ, respectively, without acyclovir, whereas these numbers were significantly lower in the presence of acyclovir (2.3, 2.5, and 1.7, respectively) (Figure 4B).

For the IFNs in static conditions (Figure 4D), similar tendencies were observed. At 2 h, both IFNα, IFNβ, and IFNλ were detected in levels similar to the control (1.3, 1.6, and 1.4, respectively). After 48 h, IFN levels increased as expected based on the studies in perfusion, but to much higher levels (27, 30 and 27, respectively) (Figure 4D). With acyclovir, a level of 1.1 was observed for all three IFNs after 48 h, which was slightly lower than in the microfluidic chip (2.3, 2.5, and 1.7, respectively). A reason for the larger quantification of IFNs in the static setup compared to the chip with perfusion could be related to the continuously perfusing medium resulting in some level of ‘clearance’ of the already secreted IFNs, which would normally induce the production and secretion of additional IFNs. However, the complex interplay between the IFN pathway and HSV, where the ICP genes disrupt the IFN response (both by blocking pathways and downregulating the level of expression of IFN-stimulated genes) [46], should also be taken into consideration.

### 3.5. Infection Gradient

**Figure 5:**
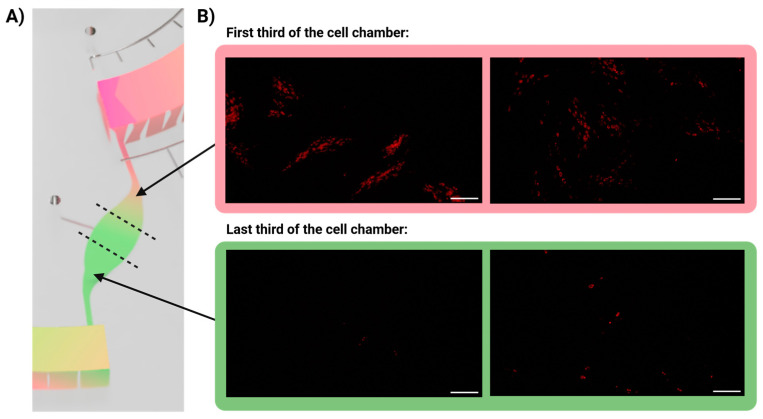
HSV-1 infection gradient occurring gradually from the inlet in the cell chamber towards the outlet from the cell chamber 24 h after infection. (**A**) Schematic overview of the areas defined for imaging. (**B**) Fluorescent images of PDL cells in different parts of the cell culture chamber infected with HSV-1 mCherry (MOI 6) at 24 h post infection. All scale bars represent 200 µm.

It was of major interest to investigate how the infected cells were distributed within the cell culture chamber during the infection in perfusion (Figure 5). Since the HSV-1 virus was introduced to the cells via medium perfusing from the first part of the cell culture chamber towards the end of the chamber, it could theoretically result in an infection gradient. For fluorescent image analysis, we considered two parts of the cell chamber, namely (i) the first third of the cell chamber (closest to the inlet channel) and (ii) the last third of the cell chamber (closest to the waste chamber) (Figure 5A).

As seen from Figure 5, an infection gradient was observed 24 h post infection, shown here as infected cells (fluorescent signal) in the first third of the cell chamber in contrast to no or very few infected cells in the last third of the cell chamber (Figure 5B). The infection gradient phenomenon was observed in the majority of the replicate studies. A gradient of infection in the cell chamber would mean that a first round of infection is occurring in the cells closest to the inlet channel (initially exposed to HSV-1) during the first 24 h, whereafter the cells in the rest of the cell chamber are infected within the next 24 h (Figure 3B). This may be explained by the fact that infections were performed at low MOI (<10), with a short exposure time (90 min), followed by washings. Moreover, the flow rate was rather low (0.24 µL/min), meaning that only approx. 20 µL of the ~3 mL medium in the medium chamber reached the cell culture chamber before washing. In such experimental conditions, it is likely that the few viral particles slowly entering the cell chamber will be trapped by the first rows of cells, leaving the cells most distant from the entrance to the chamber uninfected. Such an infection gradient has not previously been reported, most likely since viral infection studies are traditionally carried out in static conditions, and, thus, all the cells are exposed to the virus at the same time. Of note, the infection experiments were only carried out with a low MOI (<10) to avoid saturating the cells with a high viral load. However, as a future perspective, it would be interesting and relevant to investigate whether the infection gradient varies with the titer of virus loading (i.e., MOI level).

## 4. Conclusions

In the present work, we have developed a flow-based in vitro infection model, based on a novel microfluidic platform, to study dynamic interactions related to oral diseases, which is otherwise not available today. We demonstrated that it is possible to seed and culture human primary PDL cells under continuously perfusing medium (0.23 ± 0.14 µL/min) and obtain a confluent cell layer. Additionally, we showed that HSV-1 replicates in PDL cells under perfusion and that the model can be used to study the effect of antiviral drugs in perfusion, with acyclovir as a proof-of-concept drug. In general, similar HSV-1 gene expressions were observed when comparing the infection of the PDL cells in the microfluidic chip to cells cultured in static conditions. However, differences in the innate antiviral response could be observed as much higher levels of IFNs in static conditions (27–30), compared to in the chip in perfusion (7.0–7.2), although the higher levels in static conditions also resulted in larger standard deviations.

Finally, we showed that the infection occurs as a gradient in the direction of the flow at 24 h before infecting cells in the entire cell chamber at 48 h. This is especially interesting since an infection gradient enables studies of intercellular communication between infected cells and cells that have not yet been infected. For future work, this would also be interesting to study in shorter time intervals. However, this would require further optimization of sampling techniques and quantification methods.

In conclusion, the developed microfluidic chip with primary human PDL cells revealed interesting differences from static experiments and offers a more physiologically relevant method to study virus infections and drug candidates in vitro. Although centrifugal microfluidics introduces additional forces compared to traditional laminar microfluidic systems, such as centrifugal, Coriolis, and Euler forces, which may result in Dean flow in the channels, our study establishes the usefulness and feasibility of this technology for cell culture, in particular for dynamic viral infections. This can pave the way for real-time evaluation of antiviral drugs, resulting in more relevant determination of the dose-response curves and EC50 values of different compounds against viral infections. Ultimately, more physiologically relevant in vitro models can lead to new proposed treatment strategies—both for periodontitis but also for other diseases caused by dysbiosis in the local microbiome, including viruses.

## Figures and Tables

**Table 1:** List of primers.

	Forward Primer (5′ → 3′)	Reverse Primer (5′ → 3′)
GAPDH	GGTGGTCTCCTCTGACTTCAACA	GTTGCTGTAGCCAAATTCGTTGT
ICP0	GTCGCCTTACGTGAACAAGAC	GTCGCCATGTTTCCCGTCTG
ICP4	CGACACGGATCCACGACCC	GATCCCCCTCCCGCGCTTCGTCCG
ICP8	CGACAGTAACGCCAGAAG	GGAGACAAAGCCCAAGAC
IFNα	AGAAGGCTCCAGCCATCTCTGT	TGCTGGTAGAGTTCGGTGCAGA
IFNβ	CTTGGATTCCTACAAAGAAGCAGC	TCCTCCTTCTGGAACTGCTGCA
IFNλ	AACTGGGAAGGGCTGCCACATT	GGAAGACAGGAGAGCTGCAACT

## Data Availability

Data is contained within the article or Supplementary Material.

## Supplementary Materials

The following supporting information can be downloaded at: https://www.mdpi.com/article/10.3390/bios14080401/s1, Figure S1: 3D printed stacking unit for enabling multiple replicates on one motor. (A) Multi-angle illustrations of the 3D printed stacking unit. (B) The stacking unit with multiple chips inserted. (C) Installation of the stacking unit on a spindle motor. (D) Various through-put models of the stacking unit; Figure S2: An image of a chip showing the volume markers used to calculate the flow rate of the system. The long markers are equal to 100 µL and the short markers are equal to 20 µL; Figure S3: Growth of PDL cells in static conditions (96 well plate). Representative brightfield images (10×) at (A) 0 h (immediately after seeding), at (B) 24 h and at (C) 48 h. A total of 7.7 × 104 cells/cm^2^ were seeded at 0 h. All scale bars represent 200 µm; Figure S4: Enlarged version of selected brightfield images (10×) from Figure 3 showing PDL cells in the chip after 4 days of culture (2 days culture followed by 48 h HSV-1 infection) in perfusion. (A) PDL cells without HSV-1 (control) showing a fully confluent cell layer. (B) PDL cells exposed to HSV-1. The HSV-1 infection results in morphological changes such as ballooning and detachment of dead cells (red arrows highlighting examples). Scale bars represent 200 µm.
