# Supplementary material for: Centrifugal Microfluidic Cell Culture Platform for Physiologically Relevant Virus Infection Studies: A Case Study with HSV-1 Infection of Periodontal Cells
Source: Biosensors (Basel). 2024 Aug 20;14(8):401. doi: 10.3390/bios14080401 (PMC11352947; doi:10.3390/bios14080401)
Supplement: Supplementary file 1 [file biosensors-14-00401-s001.zip › biosensors-3122187-supplementary.pdf]

Supplementary materials

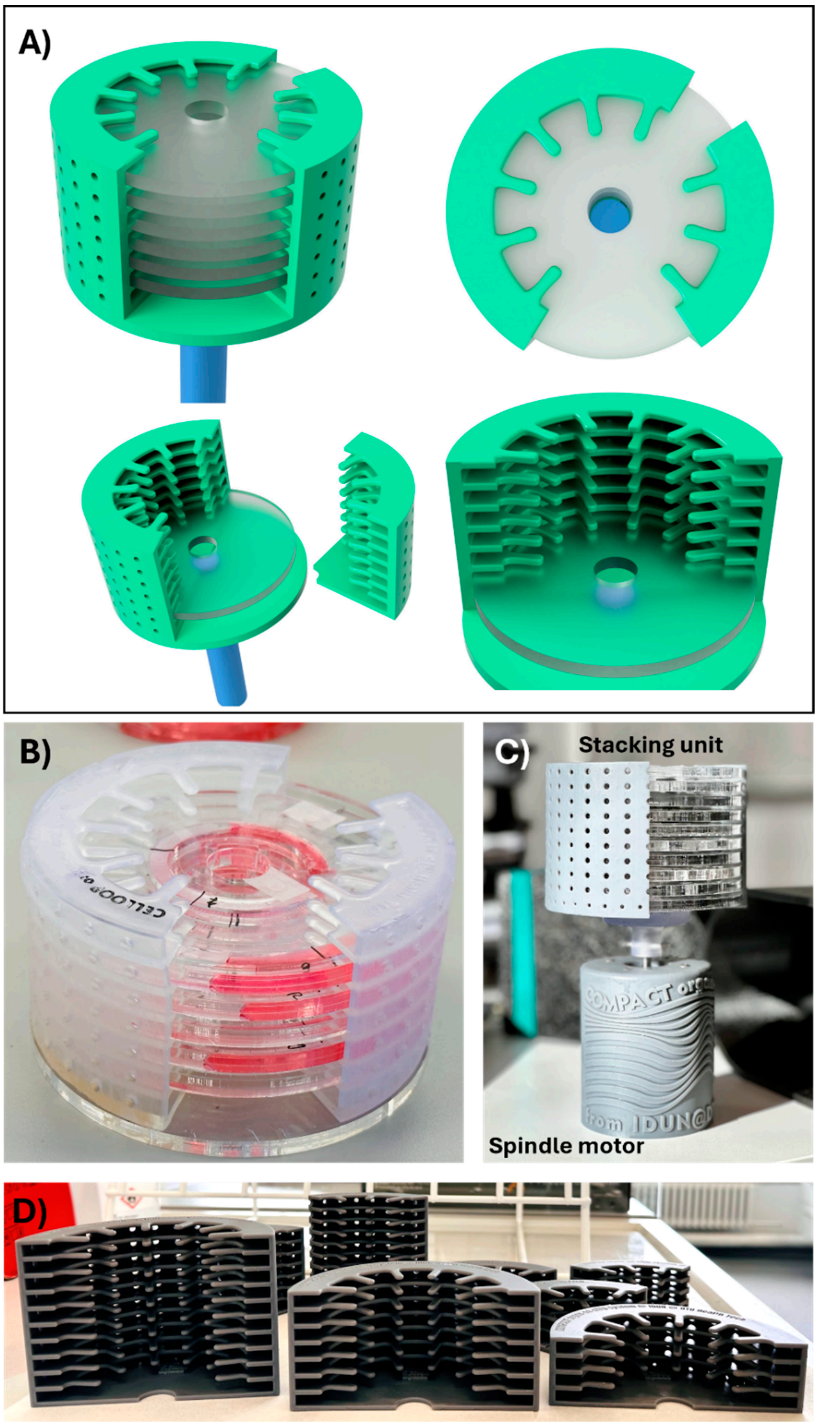

Figure S1: 3D printed stacking unit for enabling multiple replicates on one motor. A) Multi-angle illustrations of the 3D printed stacking unit. B) The stacking unit with multiple chips inserted. C) Installation of the stacking unit on a spindle motor. D) Various through-put models of the stacking unit.

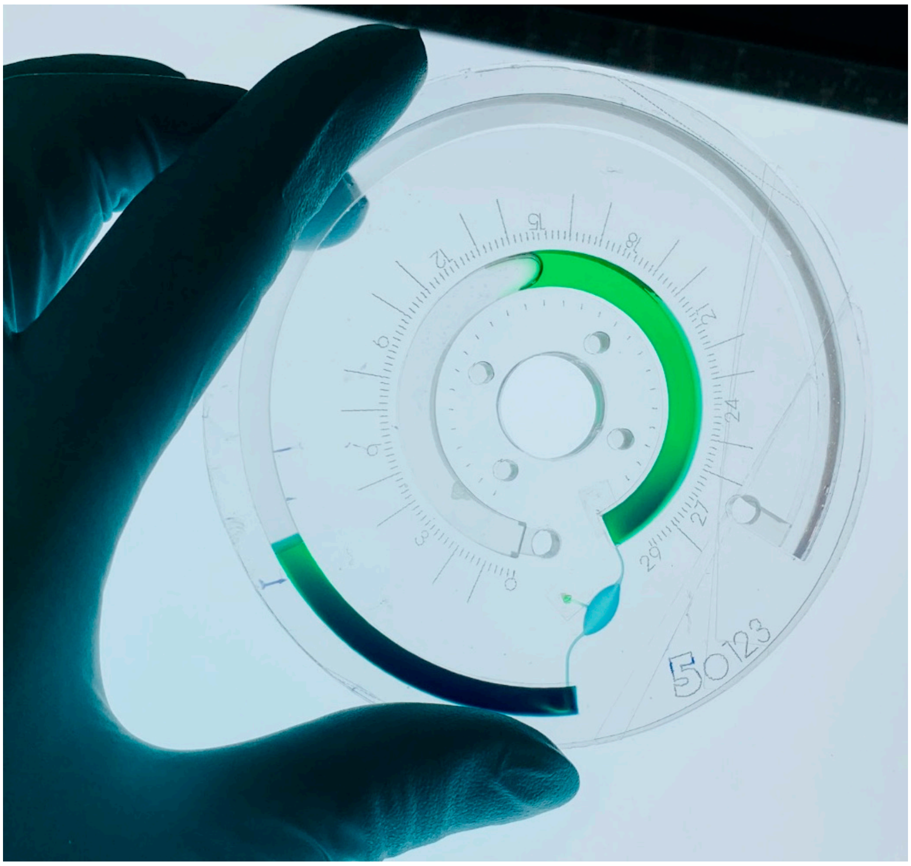

Figure S2: An image of a chip showing the volume markers used to calculate the flow rate of the system. The long markers are equal to 100 μL and the short markers are equal to 20 μL.

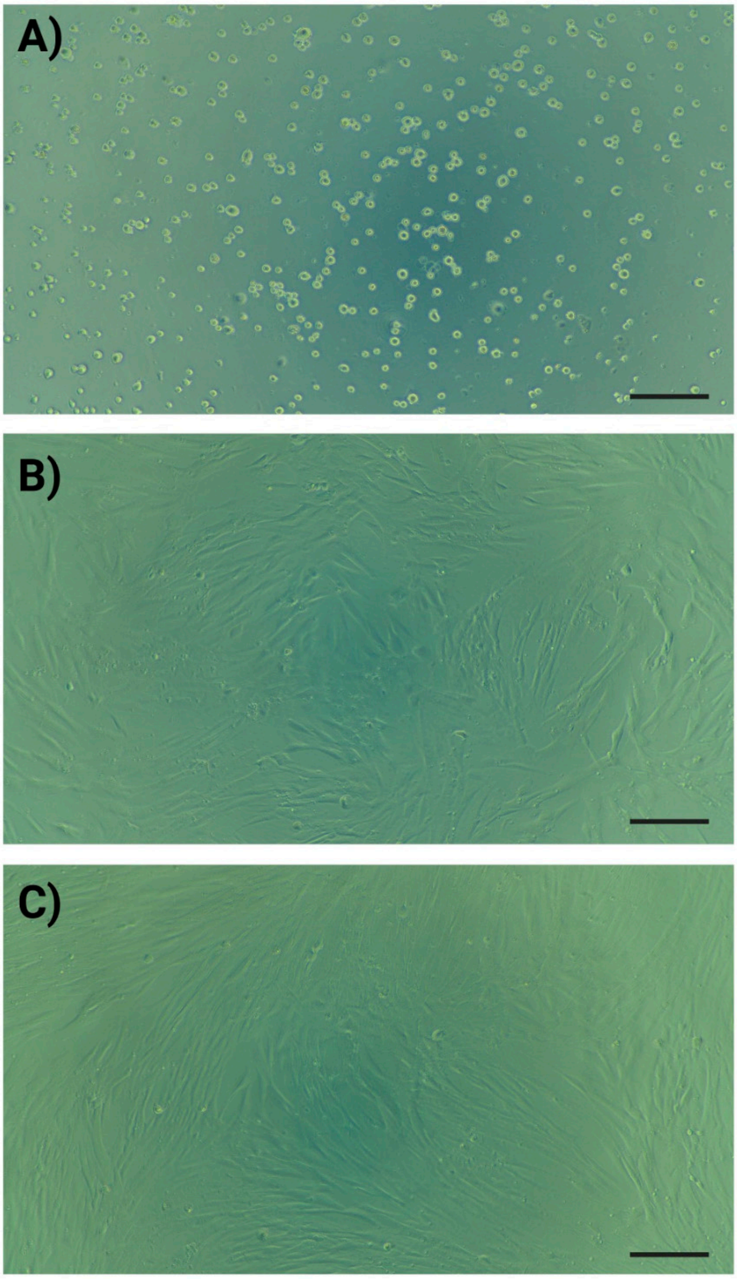

Figure S3: Growth of PDL cells in static conditions (96 well plate). Representative brightfield images (10x) at A) 0 h (immediately after seeding), at B) 24 h and at C) 48 h. A total of 7.7 x 104 cells/cm2 were seeded at 0 h. All scale bars represent 200 µm.

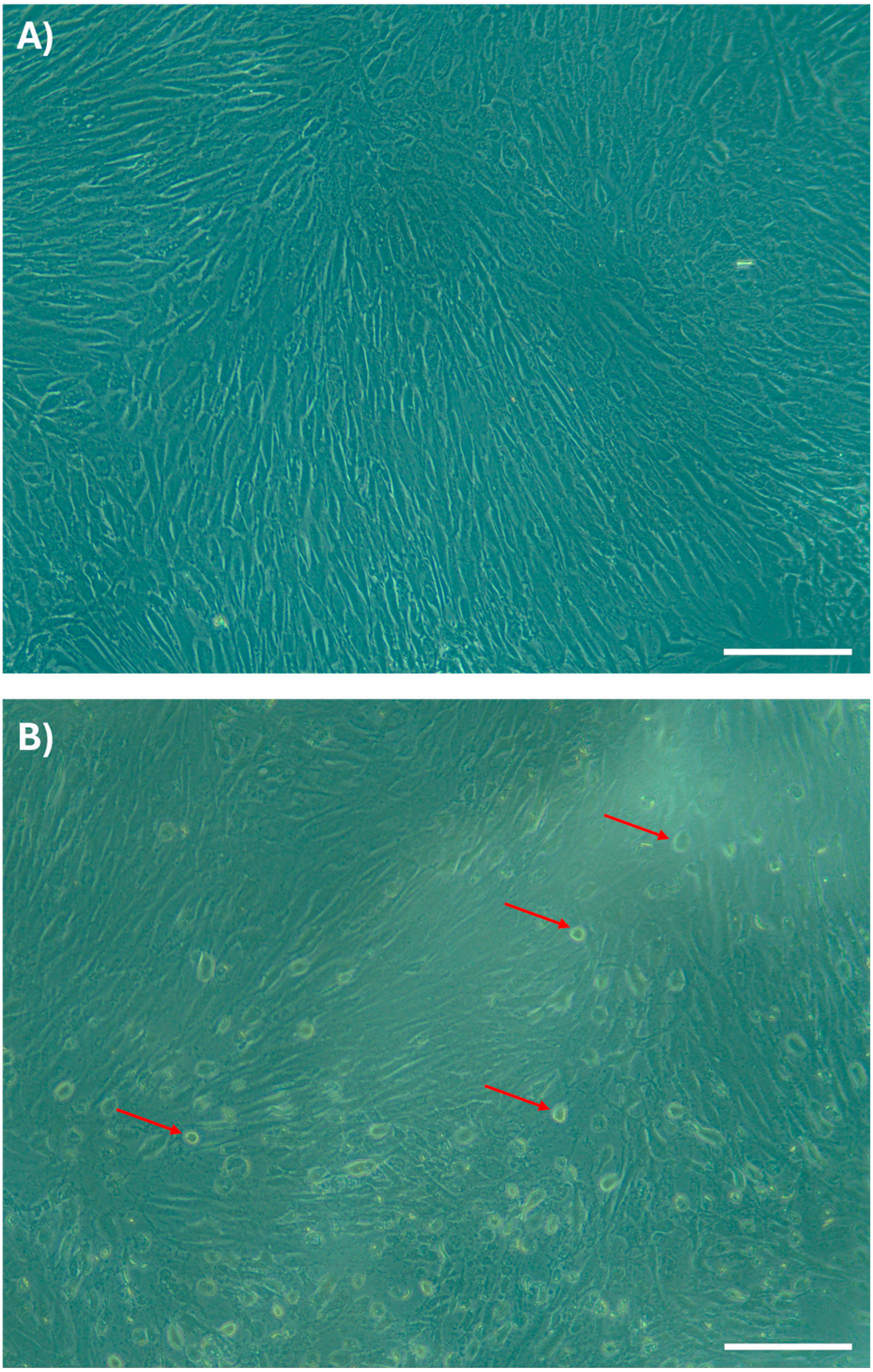

Figure S4: Enlarged version of selected brightfield images (10x) from Fig. 3 showing PDL cells in the chip after 4 days of culture (2 days culture followed by 48 h HSV-1 infection) in perfusion. A) PDL cells without HSV-1 (control) showing a fully confluent cell layer. B) PDL cells exposed to HSV-1. The HSV-1 infection results in morphological changes such as ballooning and detachment of dead cells (red arrows highlighting examples). Scale bars represent 200 μm.
